# Acquired perforating disorder in a patient with *Strongyloides stercoralis*

**DOI:** 10.1186/1471-2334-13-S1-P79

**Published:** 2013-12-16

**Authors:** Mircea Tampa, Vasile Benea, Anca Malin Benea, Cristian Oancea, Isabela Sârbu, Simona-Roxana Georgescu

**Affiliations:** 1 Carol Davila University of Medicine and Pharmacy Bucharest, Romania; 2 Clinical Hospital of Infectious and Tropical Diseases “Dr. Victor Babeş”, Bucharest, Romania; 3 Victor Babeş University of Medicine and Pharmacy, Timişoara, Romania

## Background

*Strongyloides stercoralis*, a geohelminth which affects humans, has a cosmopolitan distribution in the tropical and subtropical areas but is sporadic in temperate countries like Romania. Acquired perforating disorders are a group of uncommon skin conditions characterized by transepidermal extrusion of dermal components, usually occurring in patients with chronic renal failure and/or diabetes mellitus. It has been rarely associated with chronic B and C hepatitis, HIV, cutaneous cytomegalovirus infection, pulmonary aspergillosis, scabies and arthropod bites.

## Case report

We report the case of a 73 year-old male patient with a skin eruption consisting of multiple umbilicated small keratotic follicular or perifollicular papules with individual central keratotic plug with a generalized distribution affecting the whole body area, sparing the palms and soles, associated with mild pruritus. Multiple generalized shallow depressed scars with a crateriform aspect were also present, following resolution of the formerly described papules. The skin lesions had occurred 6 years before presentation.

Laboratory findings demonstrated severe eosinophilia. HBV, HCV and HIV testing turned out negative. The fasting blood glucose level and the glucose tolerance test were within normal range. The coproparasitologic exam revealed the presence of the rhabditoid larvae of the parasite *Strongyloides stercoralis*. A biopsy was performed from one of the lesions and the histopathologic examination revealed the presence of epidermal invaginations with central parakeratotic columns perforating into the dermis, with an accompanying granulomatous reaction and was consistent with the clinical diagnosis of perforating dermatosis.

## Conclusion

The patient was treated with oral albendazole (400 mg/day for 3 days) and clindamycin with favorable results and regression of pruritus. Various infectious disorders have been associated with acquired perforating disorders. However, this is the first reported case in which strongyloidiasis could have precipitated the perforating dermatosis, in a patient without any risk factors, who never travelled to an endemic area.

